# Immediate Mobilisation After Elective Colorectal Surgery—Patients' and Healthcare Professionals' Experiences: A Qualitative Study

**DOI:** 10.1111/scs.70072

**Published:** 2025-07-01

**Authors:** Rose‐Marie Wilnerzon Thörn, Anette Forsberg, Rebecca Ahlstrand, Agneta Anderzén‐Carlsson

**Affiliations:** ^1^ Department of Physiotherapy, School of Medical Sciences, Faculty of Medicine and Health Örebro University Örebro Sweden; ^2^ Department of Anaesthesiology and Intensive Care, School of Medical Sciences, Faculty of Medicine and Health Örebro University Örebro Sweden; ^3^ University Health Care Research Centre, Faculty of Medicine and Health Örebro University Örebro Sweden

**Keywords:** colorectal surgery, enhanced recovery after surgery, immediate mobilisation, postoperative care, qualitative study

## Abstract

**Background:**

Early mobilisation is advocated after colorectal surgery. Mobilising patients 30 min after arrival to the post‐anaesthesia care unit has been shown to be feasible; however, before implementation in clinical care, the experiences of patients and healthcare professionals must be investigated. Thus, this study aims to explore patients' and healthcare professionals' experiences of immediate mobilisation.

**Methods:**

A total of 41 patients from the intervention group of a randomised control trial investigating the effects of immediate mobilisation were included and interviewed individually between October 2017 and June 2019. Five healthcare professionals working with the intervention were also interviewed individually during fall 2018 to spring 2019. The data analyses of the two samples were conducted separately, using inductive qualitative content analysis.

**Results:**

Three themes were identified from the patients' interviews: ‘The body cannot keep up’, describing challenges, such as burdensome symptoms, reluctance and fear that mobilisation could be harmful; ‘Being in good hands’, referring to receiving individual support from healthcare professionals when being mobilised and thus feeling safe; and ‘A first step towards recovery’, describing patients' readiness for immediate mobilisation with fewer postoperative symptoms, while recognising the benefits and feeling satisfied. An overarching theme was identified from the healthcare professionals' interviews – ‘Balancing gains and challenges’, which describes likely benefits to patients but also notes that mobilisation immediately after surgery is resource intensive. The professionals described their initial concerns about patients' wellbeing and safety and the adapted facilities needed for immediate mobilisation to be smooth.

**Conclusion:**

Immediate mobilisation after elective colorectal surgery was found to be both beneficial and challenging by patients and healthcare professionals; it requires skilled staff, sufficient resources, adapted facilities, and clear mobilisation procedures and efforts tailored to the patients' individual abilities.

## 1 Introduction

Early postoperative mobilisation is considered to be an integral component of Enhanced Recovery After Surgery (ERAS) for patients going through elective colorectal surgery [1]. ERAS is a multimodal and multidisciplinary pathway (https://erassociety.org) consisting of a combination of pre‐, per‐and post‐surgical interventions for faster recovery after surgery [1, 2, 3, 4]. Early mobilisation reduces the risk of postoperative complications and the hospital length of stay, while accelerating the recovery of functional walking capacity [5]. The latest guidelines for colorectal surgery recommend that patients be mobilised out of bed on the day of surgery, at minimum by sitting in a chair for 2 h, and to be out of bed for at least 6 h daily for the rest of their stay in hospital [6].

The time point at which patients first get out of bed for mobilisation varies widely; moreover, despite recommendations, most patients in the surgical ward do not leave their bed until the day after surgery and then do so to a lesser extent than recommended [7, 8]. In a recent randomised controlled trial (RCT) [9], we explored whether immediate structured mobilisation where ‘immediate’ is defined as mobilisation starting as soon as 30 min after arrival in the post‐anaesthesia care unit (PACU) could enhance in‐hospital physical activity compared with standard care involving mobilisation starting later in the ward. The PACU in question have 13 recovery beds in an open floor plan. The results showed that immediate mobilisation was feasible and could safely be initiated in immediate postoperative care [10]. However, there were no differences between the groups regarding the results of their in‐hospital physical activity [9]. Svensson‐Raskh et al. [11] reported similar results regarding the feasibility of mobilisation within 2 h and up to 6 h postoperatively for patients after elective abdominal surgery. Their results also indicated respiratory benefits for the patients. They also investigated patients' and healthcare professionals' experiences of mobilisation within 2 h after surgery [12, 13] and found that the patients viewed mobilisation as a crucial aspect of their recovery that influenced both their physical and their mental wellbeing. The healthcare professionals observed positive benefits for patients through mobilisation without compromising patient safety. Another study examined patients' experiences with early mobilisation following a caesarean section. There, patients who have been mobilised to standing up immediately in the PACU reported higher levels of anxiety compared to those who were mobilised 6 h after surgery [14]. Additionally, a study by Huaing et al. [15] highlighted the challenges faced by elderly patients mobilised within 4 h after lumbar spinal surgery, emphasising the importance of perioperative education on early mobilisation. Such education can help reduce stress and anxiety during the recovery process.

Our approach of immediate mobilisation starting as soon as 30 min after arrival at the PACU is a radical change in clinical care from standard care, where the mobilisation starts later, on the surgical ward. Successful implementation of this new approach requires coordination and collaboration among various care disciplines and the active involvement of the patients. Therefore, understanding different perspectives is crucial for effective patient care and the practices of healthcare professionals. Thus, the aim of this study was to explore patients' and healthcare professionals' experiences of immediate mobilisation after elective colorectal surgery.

## 2 Methods

### 2.1 Design

This study is part of a larger project [9]. The study adopts a qualitative descriptive design using qualitative content analysis with an inductive approach [16, 17], as described by Graneheim and Lundman [18]. The study follows the Consolidated Criteria for Reporting Qualitative Research checklist (COREQ) [19].

### 2.2 Setting

The setting for the study was a PACU at a university hospital in central Sweden, with 13 beds and 15 healthcare professionals working in an open‐floor environment. As standard care, patients are monitored after colorectal surgery for at least 1 h and up to 3–4 h. When the patients are respiratory and circulatory stable, they are discharged to the surgical ward.

### 2.3 Immediate Mobilisation

In the RCT [9], a mobilisation protocol was used that started from no mobilisation (level 0) to ambulation (level 4) based on the Surgical ICU Optimal Mobilisation Score (SOMS) [20]. This gradual mobilisation was carried out in 30‐min intervals, with 30 min of rest between attempts during the first, second, third, and fourth hour after arrival at the PACU [10]. At each mobilisation attempt, the patients were mobilised as far as they were capable, following the steps of the SOMS, while adhering to safety criteria [21]. This continued until discharge from the PACU or for a maximum of 4 h. The first author (RM, physiotherapist) alone assisted all mobilisation until the patients were sitting on the bedside. Any mobilisation beyond this level was performed with additional help from a nurse or nursing assistant.

### 2.4 Participants

#### 2.4.1 Patients

The inclusion criterion was being randomised to the intervention group in the RCT [9] – that is, all patients randomised to immediate mobilisation were also enrolled in this study. The reason for enrolling all 72 patients was to obtain a variety of data capturing patients' unique experiences during their short time in the PACU. However, although all patients were interviewed, it later turned out that there was no interview data available for 11 patients due to technical problems with the audio recorder. In addition, when the analysis began, 20 interviews were excluded, as the participants explicitly or implicitly revealed not remembering their mobilisation at the PACU. Therefore, interview data from 41 of the 72 recruited and interviewed patients—24 women and 17 men—were analysed in the current study. The ages ranged from 56 to 78 (Table 1).

**TABLE 1:** Characteristics of the participants (patients) (*n* = 41).

Age, years	
Mean (±SD)	66 (14)
Duration of surgery, min	
Median (IQR)	176 (116–233)
Women, no. (%)	24 (59)
Physical status (ASA score), no. (%)	
Scores I–II	31 (76)
Scores III–IV	10 (24)
Surgical approach, no. (%)	
Laparoscopic/robotic	20 (49)
Open surgery	18 (44)
Conversion to open surgery	2 (5)
Through existing stoma	1
SOMS level^a^ at the PACU, no. (%)	
Level 0	0
Level 1	0
Level 2	2 (5)
Level 3	15 (37)
Level 4	24 (58)

Abbreviations: ASA, American Society of Anesthesiologists; PACU, post‐anaesthesia care unit; SOMS, (Surgical ICU Optimal Mobilisation Score) level 0 = no activity; level 1 = in‐bed activity; level 2 = sitting; level 3 = standing and level 4 = ambulating.

^a^ Highest achieved level of mobilisation during stay at the PACU.

### 2.5 Healthcare Professionals

The inclusion criterion was healthcare professionals who had been involved in the immediate mobilisation of at least two patients in the RCT. All eligible participants received written and verbal information about the study from a co‐author (AF) and were given the opportunity to ask questions about the study during a face‐to‐face staff meeting at the PACU. Of 15 potential participants (registered nurses and certified nurse assistants), five volunteered for inclusion. Of these, four were registered intensive care nurses and one was a certified nurse assistant (age: median 47 years (range 25–59); professional experience: median 25 years (range 1–44); number of years in the PACU: median 7 (range 5–27)). All participants were women.

### 2.6 Data Collection

#### 2.6.1 Patients

Data were collected by a telephone interview following a semi‐structured guide [22], conducted by the first author (RM) (Table 2).

**TABLE 2:** Interview guide.

*Patients* **Opening question** You are participating in the trial ‘immediate mobilisation after colorectal surgery’. Can you recall your thoughts and feelings about getting up as early as at the post‐anaesthesia care unit? **Questions** – Can you please tell me your experiences of being mobilised in a very early stage after surgery as early as in the post anaesthesia care unit—for example, having some movements in bed, sitting up on the bedside or even standing or walking? – What were the benefits? – What were the challenges? – Can you please tell me about your experiences regarding any feelings about safety and trust in the staff during mobilisation? – Were there any situations where you felt less secure? **Probing questions** How did you feel? What made you feel that way? Can you describe …? What happened then …? How did it affect you? You mentioned … can you explain that further? **Final question** If you were to undergo similar surgery again, is there anything you would like to be done differently regarding immediate mobilisation in the post‐anaesthesia care unit? *Healthcare professionals* **Opening question** Consider one or two study patients who received immediate mobilisation. **Questions** – Tell me about your experience of the immediate mobilisation for that particular patient(s). What problems/challenges did you encounter? How did you solve them? – Can you give examples of when it worked well/less well? Advantages/disadvantages? – Did you feel that there were risks during the mobilisation in terms of patient safety? Example(s)? – What meaning do you feel that immediate mobilisation had for the patient? – What meaning do you feel that immediate mobilisation had for you as registered nurse or nurse assistant? How did this way of working affect your work situation? How did the collaboration work in order to carry out the mobilisation? Barriers? Facilities? Did immediate mobilisation affect your or your colleague's workload? **Final question** Do you have any thoughts or reflections on how to implement this immediate mobilisation in the post‐anaesthesia care unit?

The interview guide was tested during a pilot interview, where it was determined that there was a need to clarify the context of interest for this study (the PACU) in order to guide the patients to focus on this specific timeframe and setting. The interviews started in October 2017 and ended in June 2019. All interviews were carried out after the follow‐up hospital visit, approximately 4 weeks after surgery. They lasted 5–15 min and also included questions about later phases of recovery (presented elsewhere).

#### 2.6.2 Healthcare Professionals

The healthcare professionals were interviewed face to face in a quiet room near the PACU, during or close to the time of their work shift. These interviews were conducted by a co‐author (AF) who had no previous or ongoing relation with the participants but had experience conducting interviews. The interviews were conducted during the fall of 2018 to the spring of 2019. The interviews followed a semi‐structured guide (Table 2) and lasted 10–25 min.

All interviews were digitally recorded and then transcribed verbatim. An individual not involved in the study performed the transcription, and each interview was assigned with a code. All personal data were removed at this stage.

### 2.7 Data Analysis

First, the patient interviews were analysed, followed by interviews with the healthcare professionals. Both datasets were analysed using qualitative inductive content analysis [18]. The transcripts were initially read by the first author while listening to the recorded interviews to confirm the accuracy and correct any errors in the text. Thereafter, the text was repeatedly read for familiarisation with the data. In the next step, sentences or paragraphs related to the study aim were identified, and meaning units were extracted and labelled with a code [18]. The codes were then compared; similarities and differences were analysed and described at various levels of abstraction and interpretation through categories and themes [18, 23]. We analysed the interviews on both the manifest and latent levels. In the manifest phase, the focus was on the more explicit content of the text; in the latent phase, the focus was on the underlying meaning of the text, with a greater degree of interpretation [18]. The process of analysis was done through close collaboration within the research group, which consisted of a multidisciplinary team with experience in post‐anaesthesia care and pain management, physiotherapy and rehabilitation, and nursing and qualitative research. NVivo software was used when analysing the interviews. A schematic of the process is provided in Table 3.

**TABLE 3:** An example of the analysis process (patients).

Meaning units	Codes	Categories	Theme
When you were supposed to get up, right then, I mean … one isn't completely awake	Only half‐awake	Feeling challenged	
I was wondering why, but it was part of that thing getting up	Wondering why mobilising already	Feeling reluctant	The body cannot keep up
I felt that was almost the worst part, feeling like, ‘I'm not sure if I can make it’	Fear of not being able to handle it	Being afraid	

### 2.8 Ethical Considerations

Research ethical approval was obtained by the Regional Ethics Board in Uppsala (2017/223 and 2018/491), and the research followed the ethical principles of the Declaration of Helsinki [24]. All patient participants provided written informed consent upon their inclusion in the RCT, which covered the interview study. The healthcare professionals provided written informed consent prior to the interviews. All were informed that their participation was voluntary and that they could discontinue the interview without consequences; they were also guaranteed confidentiality.

## 3 Results

Three themes were identified during the analysis of the patient data – ‘The body can't keep up’, ‘Being in good hands’ and ‘A first step towards recovery’ – illustrating the patients' various experiences of being mobilised immediately after surgery. The analysis of the healthcare professional's data resulted in one theme, ‘Balancing gains and challenges’. The results from the two populations are presented separately in the text and together in Table 4. Quotations from the participants are used to illustrate certain aspects of the findings.

**TABLE 4:** Overview of the themes and categories of experiences of immediate mobilisation.

Themes	Categories
*Patients*	
The body cannot keep up	Feeling challenged
	Feeling reluctant
	Being afraid
Being in good hands	Feeling supported
	Feeling safe
A first step towards recovery	Feeling ready
	Recognising benefits
	Feeling satisfied
*Healthcare professionals*	
Balancing gains and challenges	Likely beneficial
	Resource intensive
	Concerns about patient's well‐being
	Collaboration is crucial
	Adapted facilities are needed

### 3.1 Patients

#### 3.1.1 The Body Cannot Keep Up

The results indicate that some of the patients felt that their body was not ready for mobilisation; ‘they described feeling challenged, feeling reluctant, and being afraid’.

##### 3.1.1.1 Feeling Challenged

For some patients, getting out of bed immediately after surgery posed a challenge, as they experienced dizziness, nausea, pain and weakness. The patients expressed a fear that mobilisation might worsen these symptoms. Some patients also reported being very tired and still affected by anaesthesia.

When one … was supposed to get up, right then, well, one is a bit, one isn't completely awake. (Interview #54)

Each step of the mobilisation could require multiple attempts, and some patients described feeling frustration when their body failed them. They described how they tried to mobilise, even if they almost managed and for short moments.

I started moving, but it was minimal, I would say … yes, I was sitting for just some moments … I could not manage more than that. (Interview #14)

##### 3.1.1.2 Feeling Reluctant

Some patients reported that they initially felt reserved and had doubts about immediate mobilisation. They expressed a need to settle and wondered why they should get out of bed immediately after surgery and why this could not be postponed until they had been transferred to the surgical ward and felt better.

Well, I thought it was strange that I had to get up so soon, because it was so difficult for me. (Interview #40)

In one case, the patient explicitly expressed a desire for rest; thus this patient perceived the staff as intrusive and bothersome when trying to mobilise the patient.

##### 3.1.1.3 Being Afraid

Certain patients felt insecure about encountering symptoms such as nausea and pain during movement, along with worry regarding potential damage to the wound.

Well, as long as one knows that nothing will rupture. However, it would not, since they [the healthcare professionals], they must know, I hope … It all went well. But that's what's on your mind when, when it [the mobilisation] starts so soon …. Will it work? (Interview #5)

Some felt insecure when standing because they felt disorientated after surgery. They felt unease because they did not entirely trust their capacity and hence were afraid of not managing to rise.

It is an inherent fear, although you know you're safe, you have an inherent fear inside, ‘will I manage?’ (Interview #20)

#### 3.1.2 Being in Good Hands

The patients described that they were pleased by being well taken care of by the healthcare professionals before, during, and after the mobilisation. They described that they *felt supported* and *felt safe* throughout the entire process, despite feeling that their body was not entirely ready to move.

##### 3.1.2.1 Feeling Supported

The patients described the healthcare professionals as being very helpful, competent and caring. The patients acknowledged the benefit of assistance and being encouraged to mobilise.

So, it was valuable to be encouraged, that you [the healthcare professionals] approached me and said: ‘Now we're going to …’ Okay, then you do it. If I hadn't had that, I guess I would have stayed in bed sleeping. (Interview #44)

The patients also described how being provided with walking aids helped them when standing and ambulating, and minimised the risk of falling.

##### 3.1.2.2 Feeling Safe

The presence and constant support of the healthcare professionals made the patients feel safe despite being in a vulnerable state. The caregivers were physically close and attentive before, during and after mobilisation, and their respectful approach facilitated mobilisation. Assurances from the caregivers motivated the patients to participate in the mobilisation process.

… since you [the healthcare professionals] were by my side, I completely trusted that I was in safe hands. (Interview #17)

#### 3.1.3 A First Step Towards Recovery

Immediate mobilisation was viewed as the first step in recovery after surgery. Some of the participants *felt ready* to get out of bed. Almost all patients *recognised benefits* for themselves in their recovery, and they *felt satisfied* with how they managed.

##### 3.1.3.1 Feeling Ready

When experiencing no pain, dizziness or other symptoms hindering them from moving, the patients felt more ready to mobilise.

Yes, I was up walking … ‘how did it feel?’ … I was feeling excellent, it went very smoothly. (Interview #22)

Some patients were surprised that they were able to sit on the bedside and walk because they had expected to be more affected by surgery than they were. There were also accounts of feeling in control of their body and having the necessary resources to get up, which made participants feel like their usual selves.

##### 3.1.3.2 Recognising Benefits

The patients expressed how the immediate mobilisation made them more alert and helped them feel like it was easier to regain their health. Some reported that their previous experiences of surgery and mobilisation motivated them, and they wanted and dared to push themselves. The patients described how immediate mobilisation made them feel mentally and physically fitter, which were important aspects of their recovery that enabled them to leave the hospital as soon as possible.

I believe it's important because – partly for the healing process, that I will soon be on the move again once the surgery is done, and also for my mental wellbeing. (Interview #9)

##### 3.1.3.3 Feeling Satisfied

The patients reported being pleased with their unexpected capacity to mobilise immediately after surgery. They felt satisfied being able to stand up, trusting their body to achieve a rapid recovery. They described feeling increased self‐esteem and autonomy due to their own achievements. Some even said that they could have walked further or stayed out of bed longer.

… and then, just 20 min after you're finished [the surgery], or maybe it was 40 min after they were done, I got to stand up in a standing position. And that's not really what you would expect, so I thought, ‘wow, it's cool that it's even possible’. (Interview #60)

### 3.2 Healthcare Professionals

#### 3.2.1 Balancing Gains and Challenges

The healthcare professionals found that initiating immediate mobilisation within 30 min of arrival was a delicate ‘Balancing of gains and challenges’. This theme covers the categories *likely beneficial*, *resource intensive, concerns about patients' wellbeing, collaboration is crucial* and *adapted facilities are needed*.

##### 3.2.1.1 Likely Beneficial

The healthcare professionals expressed that mobilisation was beneficial for patients of all ages. They noted positive effects such as improved breathing, reduced fear of movement and enhanced sleep. They believed that many patients had a positive attitude to immediate mobilisation, reporting increased hope and motivation for a speedier recovery, which contributed to patients' physical and mental wellbeing.

I think many patients believe that it's positive, that is, one should be on one's feet as quickly as possible, that's always the best. One feels better that way, both physically and mentally. That is common knowledge, from being ill oneself.

(Interview #6)

##### 3.2.1.2 Resource Intensive

The healthcare professionals stated that sufficient time was needed for them to coordinate the different actions related to immediate mobilisation, which posed challenges to their work processes. They indicated that the stabilisation of a patient's reactions to surgery, such as nausea, pain, or unstable blood pressure, required their focus and time. Immediate mobilisation demanded a solution‐focused approach, coupled with effective prioritisation skills.

… Pain relief must start early … and some aren't even fully awake … ‘What is their problem, is it nausea or pain?’… So, after half an hour [I have to assess] … how much pain relief is needed … it requires a lot of structure … and planning from our point of view … (Interview #2)

##### 3.2.1.3 Concerns About Patients' Wellbeing

Some healthcare professionals expressed that immediate mobilisation was inappropriate for certain patients who needed to rest in bed to recover from the effects of surgery and anaesthesia. Furthermore, the preoperative physical status of the patients was considered to affect their postoperative wellbeing and ability to mobilise.

It depends on their preoperative status. It's mainly about that, their wellbeing and if they have been able to eat and drink, and are not dehydrated or suffer from malnutrition. If they are quite well before the operation, they usually feel well afterwards, too. (Interview #4)

The healthcare professionals stated that it was crucial to assess the patient's physical condition before initialising immediate mobilisation and that it was their role to do so. Generally, they regarded it as inappropriate to mobilise patients in need of analgesics or vasopressor support. When feeling hesitant about the appropriateness of immediate mobilisation, the healthcare professionals could sometimes insist on postponing or pausing the mobilisation, to safeguard the wellbeing of the patient.

##### 3.2.1.4 Collaboration Is Crucial

Well‐functioning collaboration was regarded as crucial, and this developed during the course of the RCT. The physiotherapist held the major responsibility for the mobilisation, supported by the nursing assistant, while the nurse was responsible for the medical aspects.

The physiotherapist is perhaps more responsible for the mobilisation, maybe [with the assistance of] a nurse and a nursing assistant because they may need some help. And the nurse takes care of medical issues. (Interview #1)

The healthcare professionals noticed that some patients were more motivated and eager to mobilise before being transferred to the surgical ward, while others lacked motivation to move at the PACU. The preoperative information—considered integral to standard care—was perceived to enhance patients' awareness of the advantages of mobilisation. It was also emphasised that the mobilisation could only proceed following consultation with the patient, thereby emphasising a collaborative approach.

##### 3.2.1.5 Adapted Facilities Are Needed

The healthcare professionals noted that immediate mobilisation in the PACU required specific adjustments to the physical environment and the availability of adequate assistive devices. However, challenges arose due to space constraints, especially when multiple professionals were simultaneously attending to patients with different needs. Maintaining patient privacy and dignity during movement was also a concern.

It has been a bit cramped … but, I must say, she [the physiotherapist] has been good at trying to put shields up in all directions, so that the patient doesn't see all the other patients … (Interview #5)

## 4 Discussion

This is the first study investigating patients' and healthcare professionals' experiences of immediate mobilisation starting as early as 30 min after arrival at the PACU. From the patient perspective, our results showed that mobilisation was challenging for some patients. Support and care from competent healthcare professionals provided the patients with a sense of trust and safety. This was regarded as a cornerstone, enabling the patients to take their first steps in recovery. The interviewed healthcare professionals identified both gains and challenges for the patients, in addition to challenges in their own daily routines when following the protocol of immediate mobilisation. Collaboration between healthcare professionals was recognised as essential, and there was a need for adapted healthcare resources and facilities.

Although immediate mobilisation in the PACU is feasible [10], our study shows that it can be experienced as a challenge for some patients, especially when experiencing symptoms such as nausea, pain and fatigue, which are common for patients after surgery [10, 12, 25]. As such, the challenges were related not only to surgery itself but also to sedation and pain medication, similar to findings regarding mobilisation in intensive care units [26]. The healthcare professionals that participated in the present study also described this issue, thereby emphasising their role in assessing the status of the patient prior to initiating mobilisation.

The need for support and assistance reported by the patients in our study consistently aligns with findings from previous studies regarding mobilisation in the context of the PACU [12], intensive care [26] and ERAS care in the surgical ward [25]. In particular, support from competent healthcare professionals was regarded as crucial for patients' sense of safety and security, which aligns with other studies [10, 12, 26]. Preoperative information about the benefits of early mobilisation proved to be important for the patients since knowledge increased their motivation. As such, our findings provide support for continued attention to the preoperative information about early mobilisation proposed by the ERAS guidelines [6]. The structured mobilisation approach using the SOMS protocol helps clarify the content of the different steps in the mobilisation process for both patients and healthcare professionals.

Immediate mobilisation represented a considerable change in care for the healthcare professionals working in the PACU. Thus, they faced challenges due to time constraints and empathised the importance of available resources for them to manage mobilisation. They also had concerns about patient safety. These results align with findings from other studies that introduced mobilisation shortly after surgery [13] and in the intensive care context [27, 28]. Those studies [13, 27, 28] reported that healthcare professionals commonly identified challenges such as staffing, expertise, concerns about patient safety and increased workload. However, the current findings also show that healthcare professionals' experiences with immediate mobilisation increased their confidence and knowledge in this regard, helping them overcome their initial concerns and become more motivated to use this approach. This shift in attitude may be related to the presence of and support from a physiotherapist, in line with results from prior studies [12].

When designing the overall project, we hypothesised that initiating immediate mobilisation in the PACU would increase in‐hospital physical activity on the ward. However, the results from our RCT showed otherwise [9]. Nevertheless, the present findings showed that some patients viewed being mobilised immediately as an initial step towards their recovery during their hospital stay, in terms of physical and mental wellbeing, consistent with findings reported by Svensson‐Raskh et al. [12]. The somewhat conflicting findings within our overall research project can be explained by the different research designs, as the interview study revealed findings not measurable in a traditional RCT. Furthermore, approximately one‐third of the participants who underwent immediate mobilisation could not recall the event. This suggests that establishing a mobilisation routine in the PACU with the aim of subsequently increasing mobilisation on the ward may not be as effective as anticipated. Further studies are necessary to enhance evidence‐based knowledge supporting immediate mobilisation, as well as to develop a model capable of facilitating its eventual implementation in the PACU environment.

The clinical implications of our study are several. First, when implementing immediate mobilisation, it is necessary to adapt the mobilisation and care according to individual needs, considering a patient's symptoms after surgery and reactions to anaesthesia. Although immediate mobilisation was feasible, it was perceived as too close to surgery by some patients and healthcare professionals. Second, support from healthcare professionals and physiotherapists is an essential aspect of care for patients to initiate mobilisation at the PACU and feel safe doing so. Healthcare professionals require knowledge and skills regarding mobilisation and access to a physiotherapist to better mobilise patients immediately after surgery. Moreover, they need adequate resources when patients are in the PACU, regardless of when patients arrive from surgery. Lastly, appropriate facilities such as enough space and equipment are needed to mobilise patients in this setting.

### 4.1 Strengths and Limitations

The sampling strategy of including all participants from the intervention group of a previous RCT proved to be a successful strategy, given the relative loss of data from patients who did not remember their mobilisation at the PACU. Another strength was the multidisciplinary researcher group, which enriched the analysis and strengthened the credibility of this study [18]. Studying the perspectives of both patients and healthcare professionals enriched the results and enabled a deeper understanding of experiences of immediate mobilisation.

There are also some possible limitations: the first author's role as both an interviewer and the physiotherapist who performed the intervention could potentially create an unequal relation, decreasing the probability that the patient participants shared both negative and positive experiences. However, the results indicated that this was not the case. Furthermore, the multi‐professional research team's involvement in the analysis prevented the first author's pre‐understanding from non‐reflexively influencing the results. One potential limitation of this study is the delay in reporting the findings. However, since the sparse literature on the topic, we believe the study contributes to the expanding literature on immediate mobilisation.

The patient participants' ability to recall their experiences of immediate mobilisation and differentiate them from later mobilisation at the surgical ward might be limited at 4 weeks after surgery. To counteract the risk of including memories of other mobilisation, follow‐up questions concentrating on mobilisation specifically at the PACU were posed. This was facilitated by the interviewer's intimate knowledge about the setting and the mobilisation procedure. Finally, the small sample size of healthcare professionals compared with the sample size of patients could be a limitation. However, the two data sets yielded approximately equal quantities of text and content, despite variations in the sample size. We do not know why only a third of all eligible health care professionals chose to participate in interviews about their experiences of immediate mobilisation. However, the findings confirm that those who participated shared both positive and negative experiences, which increases the credibility of the findings despite the small sample size.

## 5 Conclusions

Immediate mobilisation starting 30 min after elective colorectal surgery was found to be both beneficial and challenging by patients and healthcare professionals. If implemented in clinical care, sufficient resources and adequate competence should be available, and clear mobilisation procedures should be adapted to the patients' individual abilities. This study contributes valuable insights useful for an eventual implementation of immediate mobilisation within the ERAS concept in the future.

## Author Contributions

Concept/idea/research design: Rose‐Marie W. Thörn, Anette Forsberg, Rebecca Ahlstrand, Agneta Anderzén‐Carlsson. Writing: Rose‐Marie W. Thörn, Anette Forsberg, Rebecca Ahlstrand, Agneta Anderzén‐Carlsson Data collection: Rose‐Marie W. Thörn, Anette Forsberg. Data analysis: Rose‐Marie W. Thörn, Anette Forsberg, Rebecca Ahlstrand, Agneta Anderzén‐Carlsson. Project management: Rose‐Marie W. Thörn. All authors have read and approved the manuscript. Experiences of immediate mobilisation after elective colorectal surgery.

## Ethics Statement

The study was approved by the ethics review board in Uppsala, Sweden, 2017/223 and 2018/491.

## Conflicts of Interest

The authors declare no conflicts of interest.

## Supporting information


Data S1.

## Data Availability

The authors have nothing to report.
